# Blood Brain Barrier‐Crossing Delivery of Felodipine Nanodrug Ameliorates Anxiety‐Like Behavior and Cognitive Impairment in Alzheimer's Disease

**DOI:** 10.1002/advs.202401731

**Published:** 2024-07-09

**Authors:** Xiaofei He, Yuan Peng, Sicong Huang, Zecong Xiao, Ge Li, Zejie Zuo, Liying Zhang, Xintao Shuai, Haiqing Zheng, Xiquan Hu

**Affiliations:** ^1^ Department of Rehabilitation Medicine The Third Affiliated Hospital Sun Yat‐sen University 600 Tianhe Road Guangzhou Guangdong 510630 China; ^2^ Department of Rehabilitation Medicine Guangzhou First People's Hospital Guangzhou 510180 China; ^3^ School of Materials Science and Engineering Sun Yat‐sen University Guangzhou 510275 China; ^4^ Nanomedicine Research Center The Third Affiliated Hospital of Sun Yat‐sen University Guangzhou 510630 China; ^5^ Guangdong Provincial Key Laboratory of Laboratory Animals Guangdong Laboratory Animals Monitoring Institute 11 Fengxin Road Guangzhou Guangdong 510663 China

**Keywords:** Alzheimer's disease, cognitive impairment, ER UPR, nanoparticles, microglia

## Abstract

Alzheimer's disease (AD) is the most common age‐related neurodegenerative disorder leading to cognitive decline. Excessive cytosolic calcium (Ca^2+^) accumulation plays a critical role in the pathogenesis of AD since it activates the NOD‐like receptor family, pyrin domain containing 3 (NLRP3), switches the endoplasmic reticulum (ER) unfolded protein response (UPR) toward proapoptotic signaling and promotes A*β* seeding. Herein, a liposomal nanodrug (felodipine@LND) is developed incorporating a calcium channel antagonist felodipine for Alzheimer's disease treatment through a low‐intensity pulse ultrasound (LIPUS) irradiation‐assisted blood brain barrier (BBB)‐crossing drug delivery. The multifunctional felodipine@LND is effectively delivered to diseased brain through applying a LIPUS irradiation to the skull, which resulted in a series of positive effects against AD. Markedly, the nanodrug treatment switched the ER UPR toward antioxidant signaling, prevented the surface translocation of ER calreticulin (CALR) in microglia, and inhibited the NLRP3 activation and A*β* seeding. In addition, it promoted the degradation of damaged mitochondria via mitophagy, thereby inhibiting the neuronal apoptosis. Therefore, the anxiety‐like behavior and cognitive impairment of 5xFAD mice with AD is significantly ameliorated, which manifested the potential of LIPUS – assisted BBB‐crossing delivery of felodipine@LND to serve as a paradigm for AD therapy based on the well‐recognized clinically available felodipine.

## Graphical abstract

Felodipine, an L‐type calcium channel blocker, is transformed to a liposomal nanodrug and applied through a low‐intensity pulse ultrasound irradiation‐assisted blood brain barrier‐crossing drug delivery. The nanodrug treatment causes a series of positive effects against Alzheimer's disease, which results in obviously alleviated anxiety‐like behavior and cognitive impairment in 5xFAD mouse model.

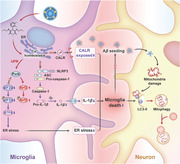

## 1 Introduction

Alzheimer's disease (AD) is the most common age‐related neurodegenerative disorder leading to cognitive impairment and dementia, it poses a significant global health challenge, which is getting worse due to aging populations.^[^ ^1a^ ^]^ Although the pathogenesis of AD has kept unclear, recent advancements in understanding AD pathophysiology have highlighted the critical role of neuroinflammation, mitochondrial dysfunction, and protein aggregation, particularly amyloid‐beta (A*β*) and hyperphosphorylated tau in AD.^[^ ^1b,c^ ^]^ Crebrospinal fluid efflux through dynamic paracellular pores on venules as a missing piece of the brain drainage system have also contributed valuable insights into these mechanisms.^[^ ^2a^ ^]^ Some Key studies have contributed valuable insights into these mechanisms, which laid down the foundation for developing innovative therapeutics.^[^ ^2b,c^ ^]^

Microglial activation takes double‐edged sword effects in the progression of AD.^[^ ^3^ ^]^ In other words, microglia clears the A*β* via endocytosis to limit the amyloid‐associated pathological changes,^[^ ^4^ ^]^ and meanwhile it activates the NLRP3 (NACHT, LRR, and PYD domains‐containing protein 3) inflammasome and thus induces a sustained innate immune response, which leads to the microglial death and release of A*β* accumulated in lysosomes into the extracellular space to promote the A*β* plaque growth.^[^ ^5^ ^]^ Furthermore, the excessive secretion of pro‐inflammatory cytokines into the neurological environment by activated microglia leads to mitochondria damage, which in turn exacerbates the inflammation to accelerate the neuronal injury.^[^ ^6^ ^]^ Even worse, the activated microglia may engulf the stressed living neurons, i.e., the so‐called microglial murder by phagocytosis.^[^ ^7^ ^]^ Despite the extensive efforts to understand the involvement of microglia in AD, the effective AD treatment based on microglia regulation is still an urgent need nowadays.^[^ ^8^ ^]^

Calcium(Ca^2+^) dyshomeostasis is critically involved in regulation of microglial function,^[^ ^9^ ^]^ it initiates the unfolded protein response (UPR) to cause endoplasmic reticulum (ER) stress for cell apoptosis, a process depending on the Perk‐eIF2*α* signaling pathway.^[^ ^10^ ^]^ Especially, under conditions of prolonged ER stress, UPR leads to a severe neurodegeneration,^[^ ^10^ ^]^ meaning that the UPR – induced ER stress could serve as a potential therapeutic target. On the other hand, Perk activation also leads to the phosphorylation of NF‐E2‐related factor 2 (Nrf2) which drives the anti‐oxidant capacity and restricts the immunostimulatory response.^[^ ^11^ ^]^ In consideration that Ca^2+^ dyshomeostasis is reversible in the early stages of A*β* pathology,^[^ ^10^ ^]^ one may reasonably deduce that a targeting therapy restoring the Ca^2+^ homeostasis is likely a potent means for treating AD.

Felodipine, an L‐type calcium channel blocker (CCB) widely used in clinic for anti‐hypertension treatment, has been found to improve the cognitive function in aged patients in a randomized and double‐blind clinical trail through an additional blood pressure‐independent effect.^[^ ^12a^ ^]^ However, oral administration of felodipine is very restricted and the bioavailability is very low because of its limited aqueous solubility and first‐pass metabolism.^[^ ^12b^ ^]^ Intravenous administration of felodipine is an alternative to oral administration and offers greater bioavailability. However, intravenous injection of free felodipine may cause severe calcium channel blocker side effects.^[^ ^13^ ^]^ Besides, repeated injection of felodipine results in transient exposures of the mice to the drug frequently, which would increase the side‐effect risks including swollen or abscesses, ankle edema, headache, flushing, dizziness, and palpitations due to the vasodilatory action of the drug.^[^ ^12^, ^13^ ^]^ Given the drawbacks of commercial product, it is of significance to develop a safer and more effective medication forms for felodipine. So far, extensive studies have verified that encapsulation in liposome, a well‐documented nanocarrier receiving intensive clinical applications, may greatly alleviate the adverse effects of therapeutic agents.^[^ ^14a^ ^]^ Furthermore, liposomes possess several characteristics such as high biocompatibility, low toxicity, ability to complex with negative and positive charged molecules, and large‐scale manufacturing.^[^ ^14b^ ^]^

**Scheme 1:**
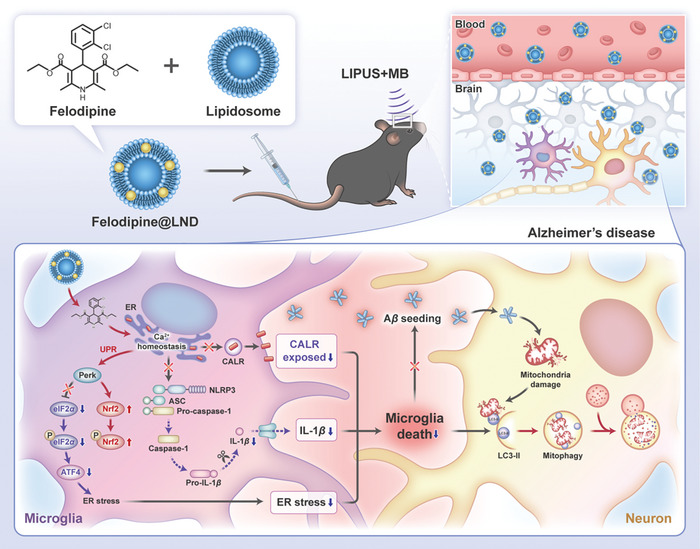
Illustration of the study design. A) Preparation and LIPUS – assisted delivery of felodipine nanodrug (felodipine@LND). B) Proposed mechanism of felodipine@LND crossing the BBB to mitigate the anxiety‐like behavior and cognitive impairment.

In spite of the high encapsulating rate of liposome nanoparticles (LNP), the blood‐brain barrier (BBB) is a major obstacle.^[^ ^15a^ ^]^ Low‐intensity pulsed ultrasound (LIPUS) induces a cavitation effect of the micron‐sized micro‐bubbles (MB) after intravenous injection, which opens the BBB for 6 to 24 h to provide a window for delivering nanodrugs into brain tissue.^[^ ^15b,c^ ^]^ Encouragingly, several clinical trials have demonstrated the safety and feasibility of ultrasound‐induced BBB opening (US – BBB).^[^ ^16^ ^]^ For example, a study recently published by Rezai^[^ ^17^ ^]^ et al. reported the use of low‐intensity ultrasound to open the blood‐brain barrier in unilateral cortex or hippocampus within 24 to 48 h, which only caused few adverse events (mainly headache) and obviously facilitated the Aducanumab penetration into brain parenchyma for reducing A*β* levels in three AD patients. Herein, aiming at ameliorating the anxiety‐like behavior and cognitive dysfunction in AD, we prepared a liposomal nanodrug incorporating felodipine (denoted as felodipine@LND) and then explored whether a transcranial LIPUS can reversibly open the blood‐brain barrier (BBB) to allow the felodipine@LND penetration for effectively treating AD in 5xFAD mouse model (**Scheme** **1**). An efficiently delivered nanodrug may hopefully switch the UPR from a pro‐apoptotic signaling toward an antioxidant signaling, decrease the A*β* seeding, and regulate the inflammatory microenvironment. If the nanodrug would work as expected, the treatment may likely promote the degradation of damaged neuronal mitochondria through unleashing mitophagy in noninflammatory conditions and improve the synaptic plasticity through reducing the loss of synapses.

## 2 Results

### 2.1 Characterization of Felodipine@LND

As shown in Figure S1A (Supporting Information), the hydrated particle size of felodipine@LND was 131.10 ± 16.39 nm, while the zeta potential was −34.02 ± 1.03 mV. As shown in Figure S1B (Supporting Information), felodipine@LND was spherical with a cavity structure of ≈100 nm. In addition, UV–vis spectrophotometry revealed the absorption peaks at 361 nm for both free felodipine and felodipine@LND (Figure S1C, Supporting Information), indicating a successful encapsulation of felodipine. The drug loading efficiency of felodipine@LND calculated by calibration curve was 4.2% and the encapsulation rate was 92.5%. In addition, the in vitro drug release behavior of felodipine@LND was investigated. As shown in Figure S1D (Supporting Information), most of the encapsulated drug was found to be released within 48 h. The release rate (concentration %) was initially fast and gradually slowed down later.

### 2.2 LIPUS Reversibly Opened the BBB to Facilitate LNP Delivery

Evans blue was intravenously injected to validate the BBB opening at different time points after an irradiation of the low‐intensity pulsed ultrasound (LIPUS) (Figure S2A, Supporting Information) following an intravenous injection of microbubble SonoVue. As shown in Figure S2B,C (Supporting Information), EB staining was clear in the brain parenchyma of mice receiving LIPUS plus SonoVue (i.e., LIPUS‐BBB group) at 0 h, whereas the contents of EB staining gradually decreased at 1 and 6 h. No appreciable EB staining was observed in the brains of mice after 24 h of LIPUS.

Two‐photon imaging showed that Coumarin 6@LNP could not permeate into the brain parenchyma for sham mice. For mice received with a transcranial LIPUS irradiation, the accumulation of Coumarin 6@LNPs in the brain parenchyma gradually increased from 1 to 10 min post‐injection (Figure S2D,E, Supporting Information). Transmission electron microscope (TEM) revealed that the integrity of vascular endothelial cells in the brain was not influenced by LIPUS (Figure S2F, Supporting Information). Moreover, Nissl's staining showed no negligible neuron degeneration, and hematoxylin and eosin (H&E) staining revealed no extravasation of erythrocytes at 24 h after LIPUS (Figure S2G,H, Supporting Information).The in vivo fluorescence imaging results showed that the DiR@LNPs were metabolized by liver in 24–48 h during circulation, both for mice with or without LIPUS‐BBB pre‐treatment (Figure S3A, Supporting Information). The LNPs could hardly accumulate in brain of mice without LIPUS irradiation. However, the DiR@LNPs permeated into the brain parenchyma of mice with LIPUS‐BBB treatment, reaching the peak at 9 h after tail vein injection and followed by a gradual decline then (Figure S3B, Supporting Information). The ex vivo results verified the brain distribution of DiR@LNPs in mice with LIPUS irradiation. Besides, there showed obvious DiR@LNPs fluorescence in liver, spleen and kidney in these two treatment groups, which may be attributed to the liver metabolism of LNPs (Figure S3C, Supporting Information). These results indicated that LIPUS combined with a commercial microbubble SonoVue can effectively and reversibly open the BBB. Notably, treatment of LIPUS plus SonoVue for BBB opening was defined as LIPUS in the following context.

### 2.3 LIPUS – Assisted Delivery of Felodipine@LND Mitigated the Anxiety‐Like behavior and Cognitive Impairment in AD

**Figure 1:**
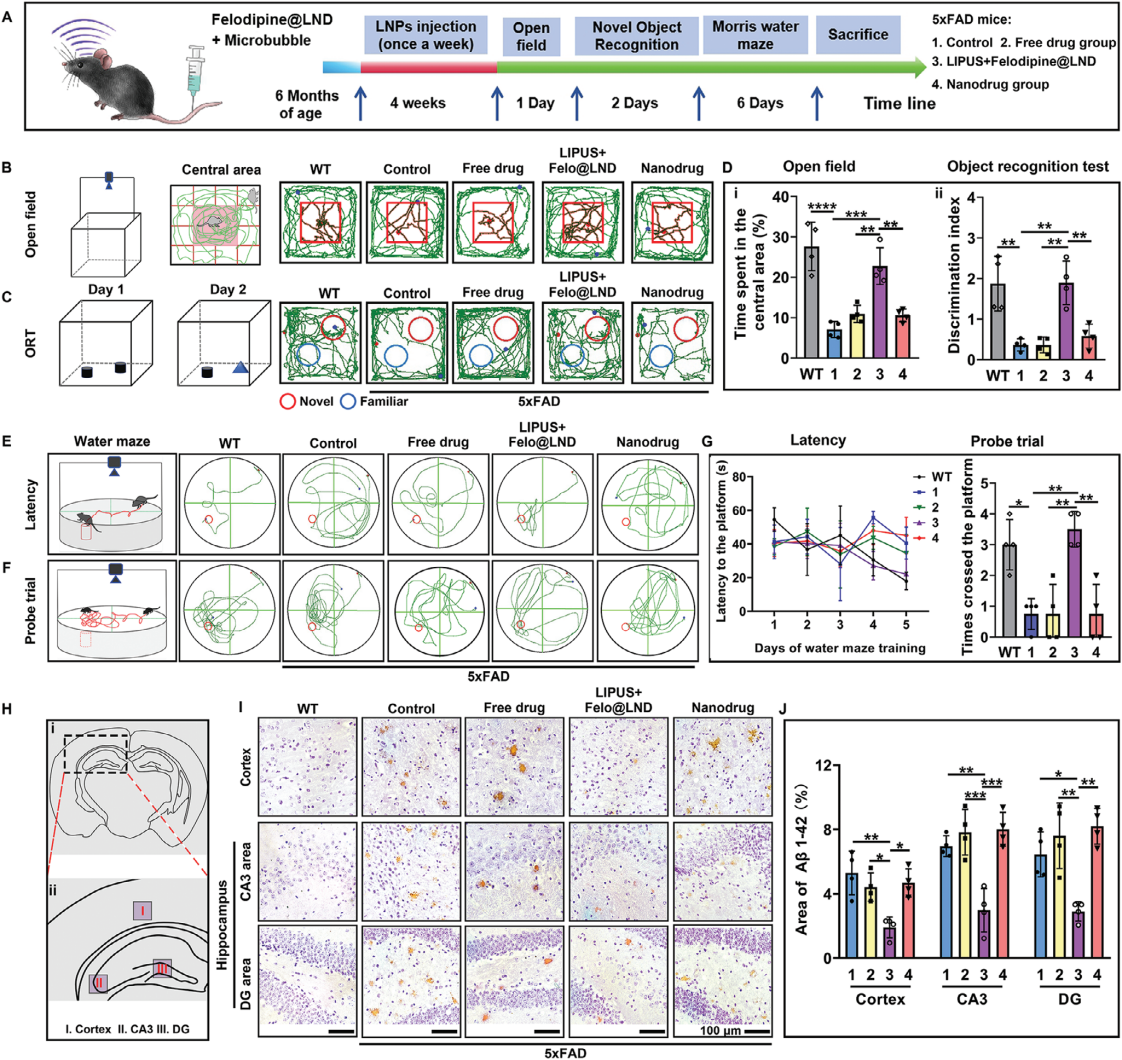
Ultrasound – assisted delivery of felodipine@LND ameliorates anxiety – like behavior and cognitive impairment. A) Experimental design for felodipine@LND administration in combination with LIPUS, Behavioral test and Pathological examination in the 5xFAD mouse model. B) Procedure diagram and travel path tracings of mice in the open field test (OFT). C) Procedure diagram and travel path tracings of mice in the object recognition test (ORT). D) The time spent in center area during the OFT (i) and the preference index in ORT (ii). E) Procedure diagram for spatial learning and travel path tracings of mice in water maze training. F) Procedure diagram for spatial memory test and travel path tracings of mice in probe trial of Morris water maze. G) The escape latency over a five‐day training course (i) and number of target crossings (ii) in the Morris water maze. H) Brain atlases indicating the cortex, dentate gyrus (DG) and CA3 areas captured in this experiment. I,J) Immunohistochemical staining and quantification of A*β*1‐42 in the cortex and hippocampus (including DG and CA3 areas) of WT and 5xFAD mice treated with PBS (Control), felodipine (Free drug), LIPUS plus felodipine@LND and felodipine@LND (Nanodrug). Data are shown as mean ± SD, *n* = 4; means ± SD; ****p < 0.0001, ***p < 0.001, **p < 0.01,*p < 0.05).

We examined the mitigated effect of LIPUS – assisted delivery of felodipine nanodrug on anxiety‐like behavior using the open field test (**Figure** **1**A,B). WT mice, without anxiety and cognitive disorder, tended to spend more time in the central area of the box. However, the 5xFAD mice received with the PBS treatment (control group) preferred staying close to the walls and travelled more time in the periphery (*p* < 0.0001) (movement toward a solid object, described as thigmotaxis), indicating apparent anxiety/threat sensitivity.^[^ ^18^ ^]^ In contrast, compared to the 5xFAD mice in the control, free drug, or nanodrug groups (Figure 1B,D‐i), 5xFAD mice received with the LIPUS plus felodipine@LND spent the most time in the central area (LIPUS+ felodipine@LND vs control, *p* = 0.0002; LIPUS+ felodipine@LND vs free drug, *p* = 0.0038; LIPUS+ felodipine@LND vs nanodrug groups, *p* = 0.0029), indicating their anxiety was significantly mitigated.

Mice have an innate preference for novelty, which is also termed as the cognitive or recognition ability. Therefore, in the object recognition test (ORT), they will tend to spend more time exploring the new object, providing information that they remember the familiar object. WT mice were more interested to the new object, showing their memory of the familiar object (Figure 1C). However, the discrimination index was significantly lower in 5xFAD mice (control group) (*p* = 0.0014), indicating the worse episodic memory. The free drug, Felodipine@LND nanodrug did not increase the discrimination index of 5xFAD mice (Figure 1C,D‐ii). In contrast, the LIPUS plus Felodipine@LND treatment significantly increased the discrimination index of 5xFAD mice (*p* = 0.0012). Morris water maze task was then performed to quantify the spatial learning and memory capability of mice (Figure 1E–G). During the training period (Figure 1E,G‐i), compared to the WT mice, the latency in 5xFAD mice (control group) on day 5 was obviously increased (*p* = 0.0084), indicating the spatial learning deficiency in AD 5xFAD mice. Compared to the control 5xFAD mice, the latency to the platform was decreased in the LIPUS plus Felodipine@LND group (*p* = 0.0381), whereas the latencies in the free drug (*p* = 0.8468) or nanodrug (*p* = 0.9356) groups did not differ from the control group (Figure 1Gi). During the probe trial (Figure 1F,G‐ii), time spent in the target area (a former platform) was significantly lower in the control 5xFAD mice compared with the WT mice (*p* = 0.0104), indicating the spatial memory impairment. The time spent in the target area was significantly higher in the LIPUS plus felodipine@LND group compared with the control group (*p* = 0.0017), but was unchanged in the free drug and nanodrug groups (Figure 1G‐ii).

We then examined the ability of felodipine@LND to reduce A*β* accumulation (Figure 1H–J). LIPUS plus felodipine@LND treatment significantly reduced A*β* plaques in the cortex (*p* = 0.0021) and hippocampus including CA3 (*p* = 0.0024) and DG (*p* = 0.0407) areas compared to the control 5xFAD mice. The free drug or nanodrug without LIPUS irradiation did not affect the A*β* plaques compared to the control group. Overall, these results indicated that the LIPUS plus felodipine@LND treatment mitigated the anxiety‐like behavior, as well as the spatial and episodic memory impairment, in AD model mice. In other words, LIPUS plus felodipine@LND is a new potential paradigm for the AD treatment, whose treatment mechanisms was detailedly investigated in the following study.

### 2.4 LIPUS – Assisted Delivery of Felodipine@LND Switched the Signaling Axis from Perk‐eIF2*α* to Perk‐Nrf2 and Reduced Endoplasmic Reticulum Stress

**Figure 2:**
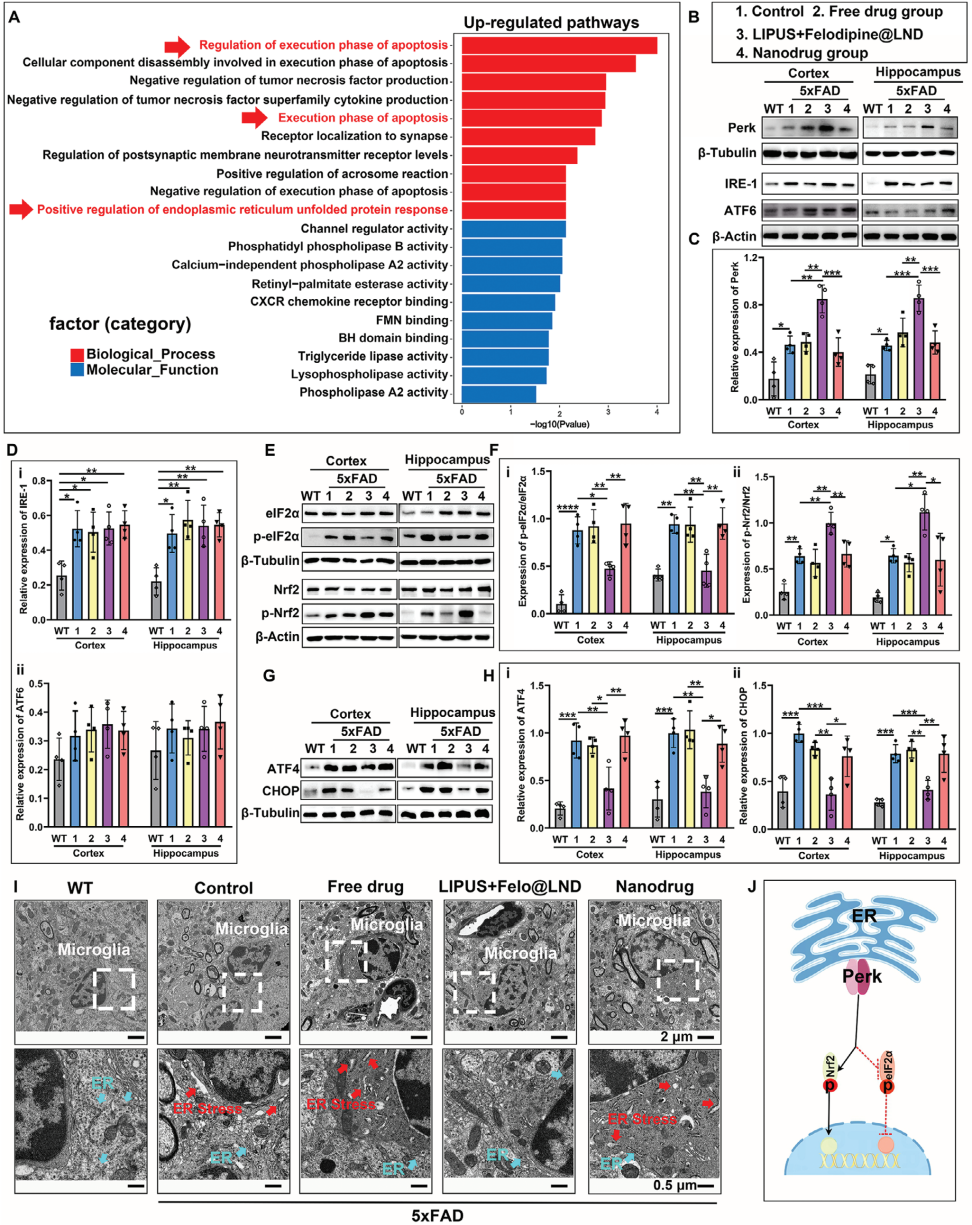
Ultrasound‐assisted delivery of felodipine@LND switched the perk‐eIF2*α* to perk‐Nrf2 signaling involving in ER UPR in microglia. A) Gene ontology (GO) enrichment analysis for DEGs involved in up‐regulated pathways for differentially expressed genes (DEGs) between control and LIPUS plus felodipine@LND groups in 5xFAD mice. B) Representative blots for Perk, IRE‐1, ATF6 levels from cortical and hippocampal tissue slice derived from WT mice and 5xFAD mice treated with either PBS (Control), felodipine (Free drug), LIPUS plus felodipine@LND and felodipine@LND (Nanodrug). C) Quantitative analysis of Perk level. D) Quantitative analysis IRE‐1 (i), ATF6 levels(ii). E) Representative blots of eIF2*α*, p‐eIF2*α*, Nrf2 and p‐Nrf2 (at Ser344) in cortex and hippocampus of WT mice and 5xFAD mice treated with either PBS (Control), felodipine (Free drug), LIPUS plus felodipine@LND and felodipine@LND (Nanodrug). F) Quantitative analysis of p‐eIF2*α*/eIF2*α* (i), p‐Nrf2/Nrf2 (ii) levels. G) Representative blots of ATF4, CHOP in cortex and hippocampus of WT mice, and 5xFAD mice treated with PBS (Control), felodipine (Free drug), LIPUS plus felodipine@LND and felodipine@LND (Nanodrug). H) Quantitative analysis of ATF4 (i) and CHOP (ii) levels. I. Representative images of TEM showing the microglia ER stress (upper panel: magnification 1.2kx; below panel: magnification for white box area in upper panel). J) Schematic illustration of felodipine@LND shifting Perk/eIF2*α* to Perk/Nrf2 arm in microglial ER UPR. Data are shown as mean ± SD, *n* = 4; means ± SD; ****p < 0.0001, ***p < 0.001, **p < 0.01,*p < 0.05).

To investigate the mechanisms mediating the described behavioral effects of felodipine@LND, RNA sequencing was performed using the Illumina Novaseq 6000 platform to investigate the differentially expressed genes (DEGs) in 5xFAD mice between the control (PBS treatment) and LIPUS plus felodipine@LND groups (**Figure** **2**A). The Gene Ontology (GO) and pathway enrichment analyses were carried out to determine the functions and pathways of DEGs. The up‐regulated DEGs in the LIPUS plus felodipine@LND group mainly involved in the regulation of the execution phase of apoptosis, receptors localized to synapse and positive regulation of endoplasmic reticulum (ER) unfolded protein response (UPR) (Figure 2A).

UPR is activated by ER under the conditions of cellular stress, which aims to re‐establishing the ER homeostasis and promote survival, but when ER stress is too severe, the UPR turns from a pro‐survival pathway into a pro‐death pathway.^[^ ^19^ ^]^ We then examined the activity of the ER UPR in the brains of control 5xFAD mice and 5xFAD mice receiving LIPUS plus felodipine@LND. Compared to WT mice, relative expressions of Perk were significantly increased in control 5xFAD brains, both in the cortex (*p* = 0.022) and hippocampus (*p* = 0.0254). The Perk level was further increased in the LIPUS plus felodipine@LND group (LIPUS + Felodipine@LND vs control group, *p* = 0.0016 for cortex, *p* = 0.0003 for hippocampus), but mice receiving the free drug or the nanodrug were unaffected (Figure 2B,C). Compared to the WT mice, the relative expression of IRE1 was also increased in the control 5xFAD mice (*p* = 0.0128 for cortex; *p* = 0.0139 for hippocampus) and 5xFAD mice receiving different treatments (*p* = 0.0232, *p* = 0.0123, *p* = 0.0065 for cortex; *p* = 0.0015, *p* = 0.0040, *p* = 0.0034 for hippocampus) (Figure 2B,D‐i). ATF6 levels showed no difference among these groups, both in the cortex and the hippocampus (Figure 2B,D‐ii). These results indicated cellular Perk and IRE1signaling was activated in the brains of AD model mice. Furthermore, while ultrasound‐assisted delivery of felodipine@LND activated Perk signaling, it did not affect the IRE1 and ATF6 signaling.

We then examined the effect of LIPUS plus felodipine@LND on the downstream response of Perk signaling. Western blots showed that p‐eIF2*α* expression was significantly higher in control 5xFAD mice than that in WT mice (*p* < 0.0001 for cortex; *p* = 0.0010 for hippocampus). Compared to the control 5xFAD group, p‐eIF2*α* expression was significantly lower in the LIPUS plus felodipine@LND group, both in the cortex (*p* = 0.0166) and hippocampus (*p* = 0.0024). However, p‐eIF2*α* expression in free drug and nanodrug groups was similar to that in the control group (Figure 2E,F‐i). Compared with the WT mice, p‐Nrf2 levels were higher in the control 5xFAD group (*p* = 0.0034 for cortex; *p* = 0.0162 for hippocampus) and further increased in the LIPUS plus felodipine@LND group (*p* = 0.0069 for cortex; *p* = 0.0112 for hippocampus). Expressions of p‐Nrf2 in the free drug and nanodrug groups did not differ from that in control 5xFAD mice (Figure 2E,F‐ii). The expressions of ATF4 and CHOP were significantly increased in control 5xFAD mice compared with the WT mice (ATF4 level: *p* = 0.0001 for cortex, *p* = 0.0007 for hippocampus; CHOP level: *p* = 0.0004 for cortex, *p* = 0.0001 for hippocampus), but were remarkably decreased in the LIPUS plus felodipine@LND group compared with the control 5xFAD mice, in both the cortex (*p* = 0.0046 for ATF4 level; *p* = 0.0003 for CHOP level) and hippocampus (*p* = 0.0023 for ATF4 level; *p* = 0.0031 for CHOP level). Expressions of ATF4 and CHOP in the free drug and nanodrug groups did not differ from those in control 5xFAD mice (Figure 2G,H). TEM results showed the normal substructure of ER in microglia for WT mice. However, expansion of the ER volume in microglia in control 5xFAD mice indicated a phenotype change under ER stress (Figure 2I).^[^ ^20^ ^]^ This change was not affected by the free drug and nanodrug without LIPUS irradiation. Fortunately, the expansion change of ER structure was ameliorated by the treatment of LIPUS plus felodipine@LND. These results indicate the LIPUS plus felodipine@LND treatment facilitated felodipine delivery into brain to shift the cellular signaling in ER URP from the Perk‐eIF2*α* axis to the Perk‐Nrf2 axis, thus resulting in an alleviation of the anxiety‐like behavior, and an improve cognition ability of mice (Figure 2J).

### 2.5 LIPUS – Assisted Delivery of Felodipine@LND Prevented the Surface Translocation of ER Calreticulin and Decreased the A*β* Plaque Growth

**Figure 3:**
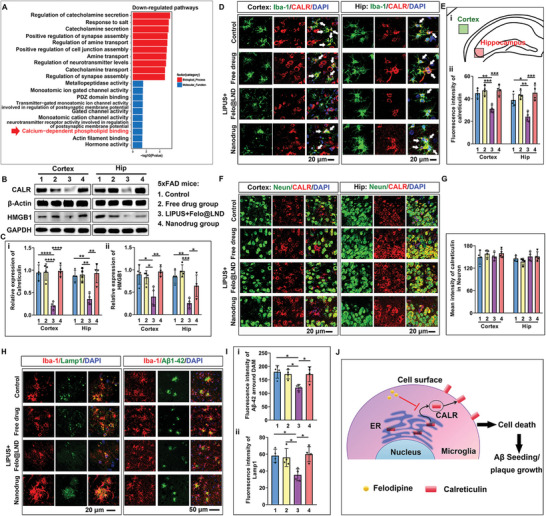
Ultrasound‐assisted delivery of felodipine@LND inhibited the cell surface translocation of calreticulin (CALR) in microglia. A) Gene Ontology (GO) enrichment analysis highlights down‐regulated pathways in differentially expressed genes (DEGs) between Control and LIPUS plus felodipine@LND groups. B,C) Representative blots and quantitative analysis of CALR and HMGB1 in the cortex and hippocampus of 5xFAD mice treated with PBS (Control), Felodipine (Free drug), LIPUS plus felodipine@LND and felodipine@LND (Nanodrug). D) Immunofluorescent images for CALR expression in Iba‐1 microglia. E) Brain atlases (i) and the quantitative analysis of microglial CALR in cortex and hippocampus (Boxes in i) in 5xFAD mice. F) Immunofluorescent images for CALR expression in neurons. G) Quantitative analysis of neuronal CALR in cortex and hippocampus in 5xFAD mice. H) Immunofluorescent images for Lamp1 in microglia (Left panel) and A*β* deposition around the disease‐associated microglia (DAM). I) Quantitative analysis of Lamp1 (i) and A*β* surrounding the DAM (ii) in cortex in 5xFAD mice. J) Schematic illustration of felodipine@LND inhibiting the surface translocation of CALR from the endoplasmic reticulum (ER) in microglia, thereby preventing microglial death and subsequent A*β* seeding and plaque growth (By Figdraw). Data are shown as mean ± SD, *n* = 4; means ± SD; ****p < 0.0001, ***p < 0.001, **p < 0.01,*p < 0.05).

GO enrichment analysis also revealed that the LIPUS plus felodipine@LND treatment down‐regulated the genes associated with the calcium‐dependent phospholipid binding (**Figure** **3**A). Calreticulin (CALR) binds to phospholipids, operating as an endogenous activator of immune cells, which participates in UPR, apoptosis, and immune response.^[^ ^19^, ^21^ ^]^ Therefore, we examined the expression of CALR to further investigate the regulation of felodipine@LND on microglial ER UPR. Western blots results showed that the relative CALR significantly decreased in the LIPUS plus felodipine@LND group, both in the cortex (*p* < 0.0001) and hippocampus (*p* = 0.0033) (Figure 3B,C), compared to the control 5xFAD group. The relative expressions of CALR had no significant difference among the control, free drug, and nanodrug groups. Specifically, immunofluorescent staining revealed that the fluorescent intensity of CALR in microglia was decreased in the LIPUS plus felodipine@LND group compared with the control 5xFAD group (*p* = 0.0028 for cortex; *p* = 0.0109 for hippocampus) (Figure 3D,E), whereas the fluorescent intensity of CALR in neurons was not different among these four groups (Figure 3F,G). Furthermore, quite a bit of CALR proteins in control, free drug, and nanodrug groups located on the surface of microglia, whereas, in the LIPUS plus felodipine@LND group, most of CALR located inside cytoplasm. As soluble and highly conserved Ca^2+^‐binding protein in the lumen of the ER, CALR actively translocates to the outer leaflet of the plasma membrane depending on the Perk‐mediated eIF2*α* phosphorylation.^[^ ^22^ ^]^ Translocation of CALR operates as a damage‐associated molecular pattern (DAMP) leading to cell death. Therefore, we examined the levels of high mobility group protein B1 (HMGB1), a maker of neurotoxic inflammatory factors released from the dying or injured cells.^[^ ^23^ ^]^ As shown in Figure 3B,C‐ii, HMGB1 levels was also decreased in the LIPUS plus felodipine@LND group as compared to the control 5xFAD group (*p* = 0.0121 for cortex; *p* = 0.0017 for hippocampus), whereas the free drug and nanodrug treatments did not affect the expressions of HMGB1. These results indicated that the LIPUS – assisted delivery of felodipine@LND more effectively inhibited the surface translocation of CALR and decreased the microglial death by decreasing the Perk‐eIF2*α* signaling.

Lysosomal dysfunction in microglia causes clearance failure of engulfed A*β*, leading to the microglial death, these dying microglia release the A*β* into the extracellular space and contribute to the A*β* seeding and plaque growth^[^ ^5^ ^]^ Lysosomal accumulation is one of the lysosomal dysfunction characteristics,^[^ ^24^ ^]^ we therefore examined the lysosomal accumulation in microglia of brain after the treatment of LIPUS plus felodipine@LND using Lamp1 (Lysosome‐associated membrane protein 1) antibody, a marker for early lysosomes. As shown in Figure 3H,I‐i, numerous Lamp1‐positive vesicles accumulated in microglia in the control, free drug, and nanodrug mice. However, the microglial Lamp1‐positive vesicles were decreased in LIPUS plus felodipine@LND group (*p* = 0.0192), indicating that LIPUS plus felodipine@LND treatment decreased the lysosomal accumulation. Besides, the fluorescent intensity of A*β* around the disease associated microglia was also decreased in the LIPUS plus felodipine@LND (*p* = 0.0123) (Figure 3H,I‐ii) compared with the control 5xFAD group, indicating the inhibition of the A*β* plaque growth after the LIPUS plus felodipine@LND treatment. However, freed drug or nanodrug without LIPUS irradiation did not influence the A*β* intensity around the disease‐associated microglia. Overall, these results indicated that LIPUS – assisted delivery of felodipine@LND decreased the lysosomal accumulation, thereby decreasing the A*β* plaque growth in 5xFAD mice brain (Figure 3J).

### 2.6 LIPUS – Assisted Delivery of Felodipine@LND Inhibited the NLRP3 Inflammasome

**Figure 4:**
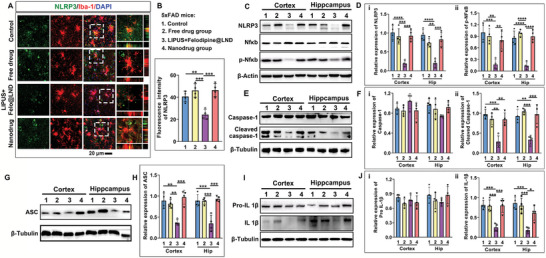
The impact of ultrasound‐assisted delivery of felodipine@LND on the inhibition of the NLRP3 inflammasome. A,B) Immunofluorescent images of NLRP3 in microglia (left panel) and quantitative analysis of NLRP3 intensity in the cortex. C) Representative blots for NLRP3 and NfκB in the cortex and hippocampus of 5xFAD mice across different treatment groups. D) Quantitative analysis of NLRP3 (i) and phosphorylated‐NfκB in the cortex and hippocampus of 5xFAD mice (ii). E,F) Representative blots (E) and quantitative analysis (F) for Caspase‐1 and cleaved Caspase‐1 in the cortex and hippocampus of 5xFAD mice across treatment groups. G,H) Representative blots (G) and quantitative analysis (H) for ASC in the cortex and hippocampus of 5xFAD mice across treatment groups. I,J) Representative blots (I) and quantitative analysis (J) for Pro‐IL 1*β* and mature IL 1*β* in the cortex and hippocampus of 5xFAD mice across treatment groups. Data are shown as mean ± SD, *n* = 4; means ± SD; ****p < 0.0001, ***p < 0.001, **p < 0.01,*p < 0.05).

Phagocytosis of A*β* has been reported to increase cytoplasmic Ca^2+^ and subsequent lysosomal damage, activating the NLRP3 inflammasome.^[^ ^10^ ^]^ NLRP3 inflammasome interacts with the adaptor protein ASC (Apoptosis‐associated speck‐like protein containing a CARD), subsequently activates the caspase‐1 and promotes the release of mature IL‐1*β* in microglia, contributing to the A*β* pathologies.^[^ ^25^ ^]^ We then examined whether felodipine@LND regulated the activation of NLRP3 inflammasomein the brain of 5xFAD mice. As shown in **Figure** **4**A,B, NLRP3 intensities in cortical microglia were decreased by the LIPUS – assisted delivery of felodipine@LND (*p* = 0.0049) (LIPUS plus felodipine@LND group), but showed no difference among the control, free drug and nanodrug groups. Compared with the control group, western blots confirmed that the LIPUS plus felodipine@LND treatment efficiently inhibited the expressions of of NLRP3, phosphorylated NFκB, cleaved caspase‐1, ASC,and mature IL‐1*β* (*p* < 0.0001, *p* = 0.0007, *p* = 0.0006, *p* = 0.0005 for cortex; *p* < 0.0001, *p* < 0.0001 *p* = 0.0013, *p* = 0.0003 for hippocampus) (Figure 4C–J).

### 2.7 Felodipine@LND Decreased the A*β* Deposition and Inhibited the NLRP3 Activation In Vitro

**Figure 5:**
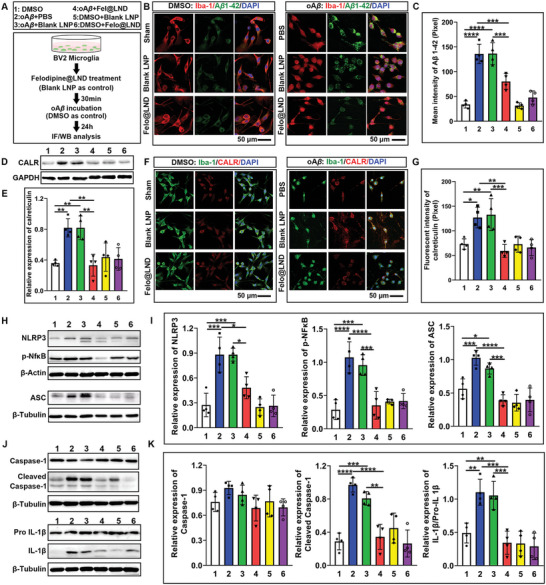
Felodipine@LND promoted the microglial degradation of A*β*, prevented the surface translocation of CALR and decreased NLRP3 inflammasome activation in BV2 microglial cell line. A) Experimental diagram to examine the microglial phagocytosis and degradation of A*β* promoted by felodipine@LND in a BV2 cell line culture. B) Immunofluorescent images showing microglial phagocytosis of A*β* plaques. C) Quantitative analysis of A*β* plaques that were not degraded by microglia. D,E) Representative blots and quantitative analysis of CALR in BV2 cells treated with or without either oA*β* plus PBS, oA*β* plus blank LNP, oA*β* plus felodipine@LND, blank LNP or felodipine@LND. F) Immunofluorescent images for microglial CALR, showing how cell surface translocation of CALR was induced by oA*β* and inhibited by felodipine@LND. G) Quantitative analysis of CALR intensities in BV2 cells. H,I) Representative blots and quantitative analysis of NLRP3 (i), phosphorylated NFκB (p‐NFκB) (ii), and ASC (iii) in BV2 cells treated with or without oA*β* plus PBS, oA*β* plus blank LNP, A*β* plus felodipine@LND, blank LNP or felodipine@LND. J,K). Representative blots and quantitative analysis of Caspase‐1 (i), Cleaved caspase‐1 (ii), Pro‐IL 1*β* and mature IL 1*β* (iii) in BV2 cells treated with or without oA*β* plus PBS, oA*β* plus blank LNP, oA*β* plus felodipine@LND, blank LNP or felodipine@LND. Data are shown as mean ± SD, *n* = 4; means ± SD; ****p < 0.0001, ***p < 0.001, **p < 0.01,*p < 0.05).

To validate the phagocytosis of A*β* and subsequent neuroinflammatory response, as well as the protection of felodipine@LND in vitro, murine BV2 microglial cells were incubated with the A*β* oligomer (oA*β*) (**Figure** **5**A). As shown in Figure 5B,C, A*β* 1–42 abundantly deposited in microglial cell in the oA*β* plus PBS group, and oAβ plus blank LNP group. However, the A*β* deposition was significantly decreased by the treatment of felodipine@LND (felodipine@LND group versus PBS group, *p* = 0.0009; felodipine@LND group versus blank LNP group, *p* = 0.0008) (oA*β* plus felodipine@LND). Western blots (Figure 5D,E) results showed that CALR level was increased in the oA*β* plus PBS group compared with the sham DMSO group (*p* = 0.0027). Compared to the oA*β* plus PBS group, the CALR level in oA*β* plus blank LNP was not different, but was decreased in the oA*β* plus felodipine@LND group (*p* = 0.0015). The change in CALR expressions was also verified by immunofluorescent staining (Figure 5F,G). CALR proteins mainly located in cytoplasm in the sham DMSO group. However, the CALR translocated to the cellular surface in the oA*β* plus PBS and oA*β* plus blank LNP groups, felodipine@LND treatment attenuated the dislocation of CALR (Figure 5F,G).

Next, we examined the attenuated effect of felodipine@LND on the activation of NLRP3 inflammasome (Figure 5H–K). The expression levels of NLRP3, p‐Nf*κ*B and ASC were significantly increased after oA*β* incubation (*p* = 0.0001, *p* < 0.0001, *p* = 0.0009). These increases were not influenced by blank LNP treatment. However, the NLRP3, p‐Nf*κ*B and ASC levels induced by oA*β* were decreased by felodipine@LND treatment (*p* = 0.0105, *p* < 0.0001, *p* < 0.0001). Without oA*β* incubation, blank LNP or felodipine@LND treatment did not affect the expressions of NLRP3, p‐Nf*κ*B, and ASC levels. The levels of cleaved caspase‐1 and mature IL‐1*β* were also increased by oA*β* incubation compared with the sham DMSO cells (*p* < 0.0001, *p* = 0.003) These levels were not influenced by blank LNP treatment, but decreased by felodipine@LND treatment (*p* < 0.0001, *p* = 0.0003, respectively). Blank LNP or felodipine@LND treatment without oA*β* incubation did not influence the expressions of cleaved caspase‐1 and mature IL‐1*β*.

### 2.8 Felodipine@LND Switched the Perk‐eIF2*α* Toward to Perk‐Nrf2 Signaling Involved in Microglial ER UPR In Vitro

**Figure 6:**
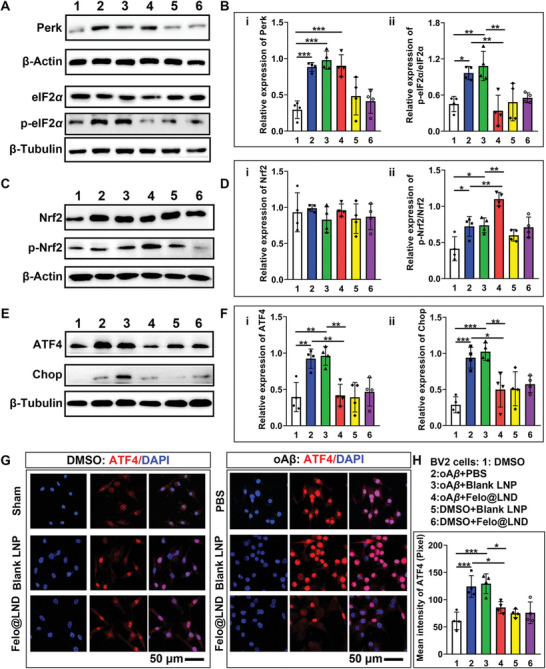
Felodipine@LND switched the Perk‐eIF2*α* toward Perk‐Nrf2 arm involved in ER UPR of BV2 microglia cell line. A,B) Representative blots and quantitative analysis of Perk, eIF2*α*, p‐eIF2*α* (Tyr150) in BV2 cells treated with or without oA*β* plus PBS, oA*β* plus blank LNP, oA*β* plus felodipine@LND, blank LNP or felodipine@LND. C,D) Representative blots and quantitative analysis of Nrf2 and p‐Nrf2 in BV2 cells treated with or without oA*β* plus PBS, oA*β* plus blank LNP, oA*β* plus felodipine@LND, blank LNP or felodipine@LND. E,F) Representative blots and quantitative analysis of ATF4 and CHOP in BV2 cells treated with or without oA*β* plus PBS, oA*β* plus blank LNP, oA*β* plus felodipine@LND, blank LNP or felodipine@LND. G,H) Immunofluorescent images and quantitative analysis for nuclear expression of ATF4. This showed the increase of ATF4 to be induced by oA*β* incubation and abolished following felodipine@LND treatment. Data are shown as mean ± SD, *n* = 4; means ± SD; ****p < 0.0001, ***p < 0.001, **p < 0.01,*p < 0.05.

We examined the signaling of ER UPR induced by oligomer A*β* (oA*β*) in vitro. Compared to the sham group, the perk levels were remarkably up‐regulated by oA*β* incubation (*p* = 0.0009); however, there was no significant difference among the oA*β* plus PBS, oA*β* plus blank LNP, and oA*β* plus felodipine@LND groups (**Figure** **6**A,B). Compared to the sham group, the phosphorylated eIF2*α* was up‐regulated in the oA*β* plus PBS (*p* = 0.0376) and oA*β* plus blank LNP groups (*p* = 0.0063), but not in the oA*β* plus felodipine@LND group. Treatment with blank LNPs or felodipine@LND without oA*β* incubation did not affect the phosphorylation of eIF2*α* (Figure 6A,B). In the cells with A*β* accumulation, the phosphorylated Nrf2 relative to total Nrf2 was remarkably up‐regulated by felodipine@LND compared with oA*β* plus PBS (*p* = 0.0065) or oA*β* plus blank LNP groups (*p* = 0.0095) (Figure 6C,D). As anticipated, western blotting results revealed that the oA*β* incubation led to an increase in ATF4 and CHOP levels compared with the sham cells (*p* = 0.0059, *p* = 0.0006, respectively). For the cells with oA*β* incubation, ATF4 and CHOP levels were not affected by blank LNP treatment compared with the oA*β* plus PBS group, but they were decreased by felodipine@LND (*p* = 0.0088, *p* = 0.0281, respectively). Blank LNP or felodipine@LND treatment without oA*β* incubation did not affect the ATF4 and CHOP levels compared with the sham group (Figure 6E,F). Besides, Immunofluorescent images verified the increase of ATF4 induced by oA*β*, as well as the regulation of felodipine@LND on ATF4 expression (Figure 6G,H), the nuclear ATF4 was increased by oA*β* Compared to the cells without oA*β* incubation (*p* = 0.0004), which was not significantly decreased in the blank LNP group but was decreased in felodipine@LND group (*p* = 0.0478). The blank LNP or felodipine@LND treatment without oA*β* incubation did not affect the nuclear expression of ATF4 (Figure 6G,H). In summary, these results demonstrated that oA*β* incubation enhanced the perk‐eIF2*α* signaling of ER UPR in microglia, thereby inducing surface translocation of CALR and activating the NLRP3 inflammasome. Felodipine@LND inhibited the dislocation of calreticulin and switched the Perk‐eIF2*α* to Perk‐Nrf2 signaling, therefore mitigating the inflammatory response in microglia and decreasing the A*β* deposition.

### 2.9 LIPUS – Assisted Delivery of Felodipine@LND Enhanced the Mitochondrial Autophagic Flux and Protected Against the Mitochondria Damage in AD Mice Brain

**Figure 7:**
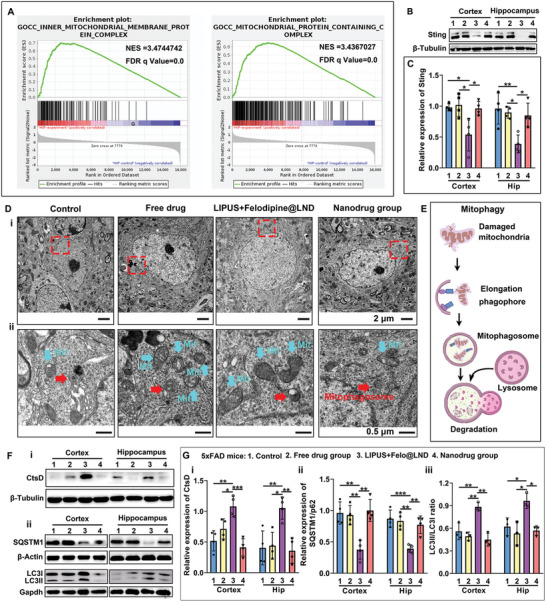
Ultrasound‐assisted delivery of felodipine@LND facilitated the autophagic flux and protected against the mitochondria damage in neuron. A). Gene Set Enrichment Analysis (GSEA) for DEGs between Control and LIPUS plus felodipine@LND groups. B,C) Representative blots and quantitative analysis of Sting in 5xFAD mice treated with PBS (Control), felodipine (Free drug), LIPUS plus felodipine@LND and felodipine@LND (Nanodrug). D) Transmission electron microscopy (TEM) images showcasing mitochondria in neurons, with (i) displaying neurons and (ii) magnifying the field labeled in the red box of panel i, with white arrows indicating mitochondria, red arrows indicating mitophagosomes, and blue arrows showing mitophagosomes undergoing degradation. E) A schematic illustration the mechanisms underlying mitophagy (By Figdraw). F) Representative blots of lysosomal cathepsin D (CtsD) (i) SQSTM1/P62 and LC3I/II (ii) in 5xFAD mice treated with PBS (Control), felodipine (Free drug), LIPUS plus felodipine@LND and felodipine@LND (Nanodrug). G) Quantitative analysis of CtsD (i), SQSTM1/P62 (ii) and LC3I/II (iii). Data are shown as mean ± SD, *n* = 4; means ± SD; ****p < 0.0001, ***p < 0.001, **p < 0.01,*p < 0.05.

Of note, Gene Set Enrichment Analysis (GSEA) for the hippocampus showed the inner mitochondrial membrane protein complex and mitochondrial protein containing complex to be up‐regulated in the LIPUS plus felodipine@LND group relative to the control group (**Figure** **7**A). In fact, proteins located in the inner and outer mitochondrial membranes cross communicate with ER Ca^2+^ signals to regulate the mitochondrial Ca^2+^ homeostasis.^[^ ^26^ ^]^ Mitochondrial Ca^2+^ overload can lead to the cytosolic release of mtDNA and activate cGAS ‐ Sting signaling, which drives aging‐related inflammation and leads to apoptosis.^[^ ^27^ ^]^ To this extent, we examined whether the Sting expressions are affected by felodipine@LND delivered by LIPUS in 5xFAD mice brain. Western blots showed that the Sting level was decreased in the LIPUS plus felodipine@LND group compared with the control, free drug, and nanodrug groups, both in the cortex (*p* = 0.024, *p* = 0.0126, *p* = 0.0289) and the hippocampus (*p* = 0.0046, *p* = 0.0010, *p* = 0.0220) (Figure 7B,C). Next, the ultrastructure of neurons was examined by transmission electron microscopy (TEM), showing the neuronal shrinkage and reduced mitochondrial cristaes in control, free drug and nanodrug groups (Figure 7D). These events are considered as the biological substrate for mitochondria damage and poor memory functioning.^[^ ^28^ ^]^ The neuronal soma did not shrink, and the mitochondrial cristaes were increased in the LIPUS plus felodipine@LND group.

Unexpectedly, the TEM results showed that the mitophagosome in neurons were detectable in 5xFAD mice brains in these different groups (Figure 7D) However, the autolysosomes undergoing hydrolysis could only be detected in the LIPUS plus felodipine@LND mice. The autolysosomes were not detected in control, free drug and nanodrug groups. This suggests that damaged mitochondria were sequestrated within autophagosomes and subsequently tagged for lysosomal degradation, an event called mitophagy (Figure 7E).^[^ ^29^ ^]^ Therefore, we examined the effect of felodipine@LND delivered by LIPUS on lysosomal proteases cathepsins D (CtsD), which is one of the major lysosomal proteases required for degrading autophagic cargoes. Consistently, CtsD expression was significantly increased in the LIPUS plus felodipine@LND group compared with the control, free drug, and nanodrug groups (*p* = 0.0020, *p* = 0.0432, *p* = 0.0004 for cortex; *p* = 0.0085, *p* = 0.0127, *p* = 0.0051 for hippocampus) (Figure 7F‐i,G‐i). Furthermore, western blot results revealed LIPUS plus felodipine@LND treatment to be associated with a significantly lower SQSTM1/P62 cellular protein concentration (*p* = 0.0018, *p* = 0.0029, *p* = 0.0010 for cortex; *p* = 0.0008, *p* = 0.0015, *p* = 0.0051 for hippocampus) and higher LC3‐II:LC3‐I ratio (*p* = 0.0068, *p* = 0.0021, *p* = 0.0010 for cortex; *p* = 0.0390, *p* = 0.0102, *p* = 0.0177 for hippocampus) compared with those in control, free drug, and nanodrug groups (Figure 7F‐ii,Gii,iii). This indicated a potential acceleration of autophagic flux.^[^ ^24^ ^]^ These results suggest that the felodipine@LND delivered by LIPUS protected against the mitochondria damage by enhancing lysosomal enzyme activity and accelerating autophagic flux.

### 2.10 LIPUS – Assisted Delivery of Felodipine@LND Decreased the Neuronal Apoptosis and Promoted the Synaptic Plasticity

**Figure 8:**
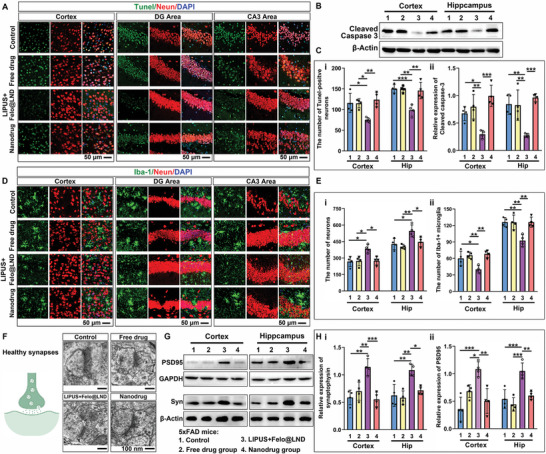
Felodipine@LND delivered by LIPUS‐BBB decreased the neuronal apoptosis and increased the synapse‐associated proteins. A) Immunofluorescent images of Tunel (Terminal‐deoxynucleoitidyl Transferase Mediated Nick End Labeling)‐positive neurons in the cortex and the hippocampus (including DG and CA3 areas). B) Representative blots against cleaved caspase‐3 for 5xFAD mice treated with PBS (Control), felodipine (Free drug), LIPUS plus felodipine@LND and felodipine@LND (Nanodrug). C) Quantitative analysis of Tunel‐positive neurons and cleaved caspase‐3 in the cortex and hippocampus. D,E) Immunofluorescent images (D) and quantitative analysis (E) of Neun positive neurons and Iba‐1 positive microglia in the cortex and hippocampus of 5xFAD mice treated with PBS (Control), felodipine (Free drug), LIPUS plus felodipine@LND and felodipine@LND (Nanodrug). F) Schematic illustration of healthy synapse (Created with gdp.renlab.cn) (i) and images of TEM to show the ultrastructure of synapses (ii) in 5xFAD mice treated with PBS (Control), felodipine (Free drug), LIPUS plus felodipine@LND and felodipine@LND (Nanodrug). G,H) Representative blots and quantitative analysis of postsynaptic density protein 95 (PSD95) and synaptophysin (Syn) in the cortex and hippocampus of 5xFAD mice treated with PBS (Control), felodipine (Free drug), LIPUS plus felodipine@LND and felodipine@LND (Nanodrug). Data are shown as mean ± SD, *n* = 4; means ± SD; ****p < 0.0001, ***p < 0.001, **p < 0.01,*p < 0.05).

We then examined the effect of felodipine@LND delivered by LIPUS on neuronal apoptosis and survival. As shown in **Figure** **8**A,C‐i, compared to the control group, LIPUS – assisted delivery of felodipine@LND was associated with significantly less Tunel‐positive neurons compared with the control group, in the cortex (*p* = 0.0199) and hippocampus (*p* = 0.0010) (including DG or CA3 areas). Free drug, and nanodrug treatments did not affect the number of Tunel‐positive neurons in either region. Additionally, LIPUS – assisted delivery of felodipine@LND decreased concentrations of the cleaved form of caspase 3, which is pro‐apoptotic,^[^ ^30^ ^]^ both for cortex (*p* = 0.0411) and hippocampus (*p* = 0.0031). However, the felodipine or felodipine@LND administration did not affect cleaved caspase 3 concentrations relative to the vehicle group (Figure 8B,C‐ii). Consistently, in the LIPUS plus felodipine@LND group, the number of neurons was increased, whereas, in both the cortex (*p* = 0.0114) and hippocampus (*p* = 0.0139), the number of microglia was decreased (*p* = 0.0413, *p* = 0.0043) compared with those in control, free drug, and nanodrug groups (Figure 8D,E). In light of this, we evaluated the architecture of synaptic terminals in cortical neurons using TEM ultra‐structure analysis (Figure 8F). This revealed that felodipine@LND delivered by LIPUS was associated with significantly more synaptic vesicles compared with the control group. Western blot assays showed that levels of synaptophysin (Syn) (*p* = 0.0010 for cortex; *p* = 0.0024 for hippocampus) and postsynaptic density protein 95 (PSD95) were increased in the felodipine@LND group (*p* = 0.0007 for cortex; *p* = 0.0009 for hippocampus), but not in the free drug and nanodrug groups (Figure 8G,H). Collectively, these results indicated that LIPUS – assisted delivery of felodipine@LND protected against the neuronal apoptosis, promoted the synaptic plasticity and decreased the microglial activation.

### 2.11 LIPUS – Assisted Delivery of Felodipine@LND was not Organ Toxic

Finally, we validated the safety of US‐assisted delivery of felodipine@LND. Hematoxylin and eosin (H&E) staining showed that the structure and morphology of the liver, heart, spleen, lung and kidney in LIPUS plus Felodipine@LND and nanodrug groups did not differ from the control or the free drug group (Figure S4A, Supporting Information). Serologic examination of hepatic and renal function using ALT (Alanine Transaminase), Albumin, ALP (Alkaline Phosphatase), total protein, AST (Aspartate Aminotransferase), D‐Bil, T‐Bil and GGT (glutamyltranspeptidase), Cre (Creatinine), UREA and UA (Uric Acid) biomarkers revealed no significant difference between vehicle and felodipine groups (Figure S4B, Supporting Information).

## 3 Conclusion and Discussion

The blood‐brain barrier (BBB) presents a major challenge for the delivery of effective therapeutics to the brain. So far, there are several promising strategies to overcome this obstacle and enhance the brain‐targeting delivery of therapeutics. For example, preclinical data suggests the therapeutics can successfully penetrate BBB via receptor‐mediated transcytosis. However, the efficiency of delivery to the brain parenchyma is still limited, especially for nanoagents,^[^ ^31a^ ^]^ which has impeded the translation of this strategy into clinical practice. Besides, engineered adeno – associated virus (AAVs) could cross BBB to deliver even large molecules to brain tissue. However, the risk of species‐specific AAVs for clinical trials still remains unknown since these vehicles behaved differently in nonhuman primates and humans.^[^ ^31b^ ^]^ On the other hand, the focused ultrasound (FUS), especially high‐energy FUS (HIFU), may induce a strong cavitation effect of microbubbles (MB) such as the clinically available SonoVue to tentatively open the BBB, which allows the delivery of therapeutic agents up to nanoscale to brain tissue of interest. However, this FUS – triggered effect of MB may cause brain injuries including mild hemorrhage, edema, or even apoptosis.^[^ ^31c^ ^]^ Herein, we employed the low‐intensity pulsed ultrasound (LIPUS), which has been widely applied in clinic, to induce the cavitation effect of microbubbles (MB) for BBB‐crossing delivery of nanodrug to brain tissue in a safe manner. Our results verified that a skull exposure of LIPUS in combination with MBs temporarily opened the BBB in a reversible way without inducing apparent adverse effects such as cell death or cerebral hemorrhage.

Upon ultrasound irradiation,^[^ ^32a^ ^]^ cavitation induced by microbubbles opens the tight junction of BBB endothelium temporarily. Within the time window of BBB opening, the nanodrug incorporating a Ca^2+^ channel antagonist felodipine (felodipine@LND) penetrates the BBB and accumulates to the brain. Then, the nanodrug can be taken up by microglia rather than neurons or astrocyte.^[^ ^32b,c^ ^]^ Once internalized into the microglia, liposome is digested with the help of lytic enzymes in lysosomal, upon which the phospholipid bilayers are broken and the encapsulated felodipine are released within the microglia in the AD brain.^[^ ^32d^ ^]^ Microglia as brain‐resident macrophages take up Aβ plaques and then transport them to lysosomes for degradation, but A*β* accumulation in lysosomes induces inflammatory responses to cause microglial death. Systematic histological and cell studies performed in our study verified that the nanodrug treatment with the aid of US plus SonoVue effectively switched the Perk‐eIF2*α* arm to the Perk‐Nrf2 arm in ER UPR, which inhibited the surface exposure of calreticulin and activation of NLRP3 to alleviate inflammatory responses in microglia as well as a protection against the mitochondria damage in neurons.

So far, monoclonal antibodies nanomedicine targeting A*β* and tau are the most promising strategy for treating Alzheimer's disease, which however requires huge expenditure. Furthermore, most clinical trials on humanized anti‐A*β* and anti‐tau monoclonal antibodies have declared failure,^[^ ^33a^ ^]^ which is usually accompanied by vasogenic oedema, cerebral amyloid angiopathy with microhemorrhages, and other adverse reactions.^[^ ^33a^ ^]^ On the other hand, siRNAs capable of effectively silencing gene expression in a sequence‐specific manner have also demonstrated great therapeutic potential for AD.^[^ ^33b^ ^]^ However, siRNA carriers have the risk of stimulating the immune response to cause some undesirable outcomes.^[^ ^33b^ ^]^ Excitingly, the felodipine nanodrug effectively mitigates the cognitive impairment and anxiety‐like behavior in a 5xFAD mouse model, which revealed the potential of our new treatment regimen in treating Alzheimer's disease. The major attraction of BBB‐crossing delivery of liposomal nanodrug incorporating felodipine to brain tissue using the LIPUS‐BBB assistance lies in the possibility of rapid transition from pre‐clinical models to patients, because the tolerability of the felodipine are known and the liposomal carrier has been used clinically.

## 4 Experimental Section

### Preparation of Felodipine‐Encapsulated LND

Lecithin, Distearoyl Phosphoethanolamine (DSPE) ‐mPEG2k, and cholesterol were purchased from Avito (Shanghai, China). Felodipine and coumarin‐6 were purchased from J&K Scientific (Beijing, China). All other reagents used in this study were commercially available at analytical grade. The felodipine nanodrug, felodipine@LND, was prepared using the thin‐film hydration method.^[^ ^34^ ^]^ Briefly, 22 mg of lecithin, DSPE‐mPEG2k and cholesterol (molar ratio of 100:10:50) and 1 mg of felodipine or coumarin‐6 were dissolved in 10 mL of methanol. Then, methanol was removed by rotary evaporation to form a lipid film, 5 ml of ultrapure water was added to dissolve the lipid film with the aid of ultrasound, the resulting lipid solution was sequentially extruded through polycarbonate membranes with pore sizes of 0.2 and 0.1 µm, respectively, to obtain felodipine@LND and coumarin‐6@LNPs. The drug loading was calculated based on the calibration curve. Specifically, free felodipine was dissolved in methanol and prepared for the stock solution at concentration of 0.5 mg ml^−1^, then the stock solution was diluted, test samples at concentration of 0.05, 0.5, 1, 5, 10, 20 µg mL^−1^ were obtained. Then, the absorbance (A) values were acquired at 361 nm on a UV–vis spectrophotometer (Lambda 950, Perkin Elmer, UK), the calibration curve of felodipine in methanol was drawn by regression analysis, and the regression equation was obtained. To determine the drug loading efficiency, the unloaded felodipine was removed using a syringe‐driven filter, and 10 mL of felodipine@LND was lyophilized, weighed, and dispersed in methanol. The absorbance (A) values were acquired and the encapsulated drug mass was calculated as W2, the administration mass of felodipine was taken as W1, the loading efficiency was calculated as W2/W1 × 100%.

In addition, the size distribution and zeta potential of the nanodrug were measured on a dynamic light scattering (DLS) instrument (BI‐PALS, Brookhaven Inst. Corp, USA). The morphology of nanodrug was observed on a transmission electron microscope (TEM, HT7800, Hitachi, Japan). To explore the cumulative release of felodipine in vitro, 1 mL of felodipine@LND solution was added to a dialysis bag (MWCO: 3500 Da) and placed into 8 mL of PBS in a 37 °C Incubator Shaker. At predesigned time points, 2 mL of solution was removed for further testing and replaced with the same volume of fresh PBS.

### Animals and Drug Treatments

The 5xFAD mice from the Jackson laboratory (Catalog NO. #034848) were bred in the Guangdong Laboratory Animal Monitoring Institute. Negative mice were taken as wild type (WT), and six‐month‐old male mice (25–30 g) were used in this study. The animals housed in cages in an air‐conditioned room were maintained on a standard laboratory diet, with food and water available ad libitum. All animal experiments were performed in accordance with the guidelines for animal experimentation of National Institute of Health guidelines and were approved by the Guangdong Laboratory Animal Monitoring Institute (A‐IACUC2022115), Guangzhou, China. Sixteen 5xFAD mice were randomly divided into four groups (*n* = 4 per group) to receive different injections of PBS (Control), felodipine (Free drug group), flodipine@LND (the nanodrug group), felodipine@LND under LIPUS (the LIPUS plus felodipine@LND group), and investigators were blinded to the group allocation during data collection. Because intravenous injection of free felodipine may cause severe calcium channel blocker poisoning,^[^ ^13^ ^]^ mice in the free drug group received intraperitoneal injection rather than intravenous injection of felodipine at 1 mg Kg^−1^ body weight.^[^ ^12^ ^]^ For the LIPUS plus felodipine@LND group, the animals were anesthetized with isoflurane delivered in a mixture of 30% O_2_ and 70% N_2_O, which was controlled by an anesthesia apparatus, the mice received intravenous (i.v.) injection of 200 µL microbubbles (MB, SonoVue, 59 mg per 5 mL in 0.9% NaCl solution), then their scalp were anesthetized with lidocaine and their skull was exposed right away to LIPUS (Intelect 2776 Mobile Ultrasound) for 1 min (Stimulus frequency 1.0 MHz, 0.5 w cm^−2^, duty cycle 50%) before the i.v. injection of felodipine@LND. Except the control group, mice in all other groups received the same felodipine dosage of 1 mg Kg^−1^ body weight per injection. All mice were injected once every 3 days for four weeks. All the mice were tolerant to the treatment, there was no exclusion in this study.

### Evans Blue Assay

The BBB opening was assessed using the evans blue (EB, MW = 960) extravasation assay.^[^ ^35^ ^]^ Admittedly, EB cannot penetrate intact BBB and thus could be used as a model drug to evaluate the BBB permeability.^[^ ^35^ ^]^ In detail, sixteen WT mice were anesthetized with isoflurane delivered in a mixture of 30% O_2_ and 70% N_2_O, which was controlled by an anesthesia apparatus, a low intensity pulsed ultrasound (LIPUS) combined with MB were applied to the mice skull at similar conditions for the felodipine@LND group. 2% EB was injected at a dose of 6 mg Kg^−1^ via tail vein at various time points of 0, 1, 6, and 24 h after LIPUS irradiation. Then, at 1 h after EB injection, mice were heart perfused with 0.9% NaCl solution, and the brains were excised for detecting the Evans blue staining levels under a microscope. The EB‐stained brains were weighed and processed into small pieces, and then 2 mL of the trifluoroacetic acid was added to extract EB for 2 h at 60 °C, and the absorbance at 540 nm was measured to quantify the amount of dye in the brain.

### Two‐Photon Imaging

To confirm BBB permeability, two‐photon imaging was performed as described previously.^[^ ^36^ ^]^ In brief, six WT mice were randomly divided into the sham group and the LIPUS‐BBB groups (*n* = 3 per group), they were anesthetized with chloral hydrate (4.2%, 0.01 mL g^−1^), LIPUS combined with MB were applied to the mice skull at similar conditions for the felodipine@LND group, then a thin cranial window over the parietal area was prepared for in vivo two‐photon imaging. Coumarin 6@LNP was injected intravenously. Subsequently, the mice were transferred to the stage of the microscope and imaging was performed with a two‐photon laser‐scanning microscope (Leica) under a water immersion objective (25X). Leakage of coumarin 6@LNP was assessed by measuring the intensity change of the coumarin 6@LNP in the extravascular compartment. Images were collected at 0, 1, 2, 5, and 10 min after the injection.

### Open Field Test (OFT)

The open field test (OFT) was used to examine the individual differences in anxiety‐like and novelty‐seeking behavior. The testing apparatus consisted of a 40 × 40 cm square arena bounded by a 40 cm wall (Xinxin Technology Co., LTD., Shanghai, China). A video camera suspended above recorded the spontaneous motor activities over 5 min trials. Thigmotaxis and time spent in the center of the arena were quantified as proxies of anxiety.

### Object Recognition Test (ORT)

After the open field test, the ORT was performed. Specifically, the mice were habituated to a blue square chamber (≈40 cm × 40 cm × 40 cm) with a white floor (Xinxin Technology Co., LTD., Shanghai, China). On the training day, the mice were placed in the open arena and allowed to explore two identical objects for 10 min (square blue blocks in 3 cm diameter). Each object was fastened to the floor, one in the northeast corner and one in the southwest corner. On test day, the object in the northeast was replaced with a novel object (red cylinder block in 3 cm diameter). The mice were permitted to investigate each of the different objects (familiar and novel) for 5 min. Each mouse's trajectory was tracked using a camera stationed directly above the test area. The cameras automatically recorded the time spent in center. The percentage time with each object was calculated. Finally, a recognition or preference index (d) could be calculated as described in a previous study,^[^ ^37^ ^]^ which was defined by the percentage of total object exploration time spent exploring the novel object.

### Morris Water Maze Test

The Morris water maze paradigm was set up as described previously.^[^ ^39^ ^]^ Briefly, during the training phase, mice conducted four trials (up to 60 s) on five consecutive training days. The latency to reach the platform during the training trials was recorded. Subsequently, the probe trial was conducted on day 6. This involved removal of the platform. Then, the mice were allowed to swim freely for 60 s. Consequently, the amount of time spent in the target area (former platform position) was recorded.

### RNA Sequencing (RAN‐Seq) Analysis

To identify the mechanism how felodipine@LND ameliorates the anxiety‐like behavior and cognitive impairment in AD, hippocampus tissue was collected after the behavioral test, and RNA sequencing (RNA‐seq) was performed by Obio Technology (Shanghai) Corp., Ltd. Specifically, six male 5xFAD mice were randomly divided into the control group (PBS injection) and the LIPUS plus felodipine@LND group (*n* = 3 per group), they were anesthetized with chloral hydrate (4.2%, 0.01 mL g^−1^) and then underwent transcardial perfusion with 40 mL of pre‐cooled 0.9% NaCl, and right hippocampus were isolated. Total RNA was extracted and mRNA was isolated using magnetic beads with oligo‐dT, the mRNA was then segmented and the cDNA was synthesized and end repaired. The cDNAs were ligated to DNA adapters and then amplified. The sequencing library was purified by magnetic beads. Qubit was used to detect the library concentration, and an Agilent fragment analyzer was used to detect the library fragment length. The Illumina Novaseq 6000 sequencing platform was performed for PE150 (Pair end 150 bp) sequencing analysis. Deferentially expressed genes (DEG) were defined as genes with a fold‐change > 1 or < −1, and a Benjamini‐Hochberg adjusted value of *p* < 0.05. R package clusterProfiler (version 3.14.3) was applied to perform the over‐representation enrichment analysis. Molecular Signature Database (MSigDB) was applied to perform the Gene Set Enrichment Analysis (GSEA).

### Transmission Electron Microscopy (TEM)

Mice underwent transcardial perfusion with 40 mL of pre‐cooled 0.9% NaCl. The left parietal cortex was obtained and kept overnight in 2% paraformaldehyde and 2.5% glutaraldehyde in 0.1 M PBS (pH 7.4). Subsequently, the samples were washed 3 times with 0.1 m phosphate buffer solution (PBS, 10 min each) and postfixed in a mixture of 1% osmic acid for 1 h at 4 °C. The samples were then washed 3 times with 0.1 m PBS (15 min per wash) and dehydrated in a gradient of ethanol solutions ranging from 50% to 100% ethanol (50%, 70%, 80%, and 90% for 10 min; 100% for 10 min 2 times). This was followed by dehydration in propylene oxide (10 min washes, 2 times), after which samples were gradually embedded with mixtures of propylene oxide and EPON812 (ratios of 3:1 for 0.5 h, 1:1 for 4 h at room temperature) before being embedded in pure EPON812 overnight. The samples were cured in the oven at 60 °C for 48 h. The polymerized samples were sectioned using an ultra‐thin microtome (Leica UC7). Then, 100 nm – thick ultra‐thin sections were prepared and stained with 1% uranyl acetate for 20 min followed by lead citrate for 12 min. Images were acquired using a Tecnai Spirit transmission electron microscope (FEI, USA). Vesicles with double‐membrane structures engulfing mitochondrial material were defined as mitophagosomes.

### Cell Culture

A murine BV2 microglial cell line was purchased from the EK‐Bioscience (Catalogue number: CC‐Y2022; Shanghai, China). The cells were cultured in DMEM/F‐12 (1:1) (Gibco, C11330500BT) containing 10% FBS and 1% penicillin/streptomycin at 37 °C in a 5% CO_2_ atmosphere. When the cells reached ≈90% confluence, they were digested with trypsin. The resultant dissociated cells were spread across 6‐well or 24‐well plates and incubated overnight. To further determine the effects of felodipine@LND on microglial phagocytosis, BV2 microglial cells were treated with PBS, blank LNP or felodipine@LND (5 µm felodipine) for 30 min and then were incubated with A*β* oligomer (oA*β*) for 24 h.^[^ ^38^ ^]^

For immune staining experiments, cells were plated onto 14‐mm coverslips at a density of 2 × 10^5^ cells mL^−1^ in a 24‐well plate, and the cells were then fixed in 2% PFA followed by 4% PFA and subjected to fluorescence microscopy. For the Western blot experiment, cells were plated at a density of 5 × 10^6^ cells per well in a 6‐well plate. The cells were then collected, and proteins were extracted for western blotting experiments.

### Amyloid Beta 1–42 (A*β*1‐42) Oligomer

A*β*1‐42 peptides were purchased from GLBiochem (Shanghai, China) Ltd (Catalog NO. P231020‐SJ052487). For A*β* 1–42 oligomer, the peptides were dissolved in hexafluoro −2‐isopropanol (HFIP) at 1 mg mL^−1^, the HFIP solution was placed in fume hood to dry, the A*β* peptides were then dissolved in DMSO by vigorous vortexing and sonication for 10 min. After adding the F‐12 buffer solution and stewing the solution overnight at 4 °C, the A*β*1‐42 oligomer was obtained from the supernatant after centrifugation at 14000 r min^−1^ for 10 min. The A*β* 1–42 oligomer were stored at −80 °C before use.

### Histology

Mice were perfused with 50 mL ice‐cold phosphate buffer saline (PBS) and left side of brains, livers, heart, spleen, lungs, and kidneys were collected and fixed in 4% (w/v) paraformaldehyde. Subsequently, they were dehydrated in 20% and 30% (w/v) sucrose. Coronal brain tissue was then sectioned using a freezing microtome (Leica, Hamburg, Germany) with a thickness of 10 µm, at intervals of 200 µm. Brain sections were boiled in citric acid buffer for 5 min in a microwave oven for immunofluorescence staining, They were then treated with 0.3% Triton X‐100 and QuickBlock Blocking Buffer (Catalog number: P0260, Beyotime Biotechnology, China)for 1 h at room temperature and incubated overnight at 4 °C with primary antibodies and secondary antibodies in PBS containing 10% normal goat serum at room temperature for 1 h.

Primary antibodies included the 1:300 Anti‐rabbit ionized calcium binding adapter molecule‐1 (Iba‐1) antibody, Wako, Japan; 1:200 Anti‐mouse ionized calcium binding adapter molecule 1 (Iba‐1) antibody, Wuhan Servicebio Technology CO., LTD., China; 1:100 Anti‐mouse purified anti‐*β*‐amyloid, 1–42 antibody, BioLegend, USA; 1:300 Anti‐mouse NeuN antibody, Millipore, USA; 1:400 Anti‐rabbit NeuN antibody, abcam, USA; 1:200 Anti‐rabbit calreticulin antibody, Abclonal, China; 1:300 FITC Anti‐rat Lamp1 antibody, abcam, USA; 1:100 Anti‐mouse NLRP3 antibody, Affinity bioscience, China; Anti‐rabbit ATF4 antibody, Affinity bioscience, #DF6008, China) Secondary antibodies included the 1:300 Anti‐mouse IgG (H+L), F(ab“)2 Fragment (Alexa Fluor 488 Conjugate), Cell signaling technology, USA; 1:300 Anti‐rabbit IgG (H+L), F(ab”)2 Fragment (Alexa Fluor 555 Conjugate, Cell signaling technology, USA; 1:300 Anti‐rabbit IgG (H+L), F(ab“)2 Fragment (Alexa Fluor 488 Conjugate), Cell signaling technology, USA; 1:300 Anti‐mouse IgG (H+L), F(ab”)2 Fragment (Alexa Fluor 555 Conjugate, Cell signaling technology, USA).

To detect neuronal apoptosis, brain slices were immunofluorescent stained for Neun and incubated with TUNEL reagent (Elabscience, China). Images were acquired using a Nikon fluorescence microscope (Nikon, Japan) or a confocal microscope (Leica, Germany). Immunohistochemical staining was performed using the Streptavidin‐HRP kit according to the manufacturer's instructions (CW2069S, Jiangsu Cowin Biotech Co., Ltd.). The primary antibody (1:100 Anti‐mouse purified anti‐*β*‐amyloid, 1–42 antibody, BioLegend, USA) was incubated at 4 °C overnight and then incubated with the secondary antibodies followed by DAB solution and hematoxylin. Hematoxylin‐eosin (H&E) and Nissl's staining were performed using a Hematoxylin‐Eosin Stain Kit (Solarbio, China) and Nissl Staining Solution (Beyotime, China).

### Western Blots

Total protein in right cortical and hippocampal tissue slices was extracted and quantified by protein extraction reagent (Thermo Fisher) and bicinchoninic acid protein assay kit (Thermo Fisher). Protein extracts were subjected to SDS PAGE gel of 7.5%, 10%, and 12.5% concentrations (Shanghai Epizyme Biomedical Technology), which were transferred to polyvinylidene fluoride membranes (Millipore). The membranes were incubated with the following primary antibodies (1:500 Anti‐rabbit Perk antibody, #AF5304, Affinity; 1:1000 anti‐rabbit IRE‐1 antibody, #DF7709, Affinity; 1:1000 Anti‐rabbit ATF6 antibody, #DF6009, Affinity; Anti‐rabbit Phospho‐eIF2*α* (Tyr150) antibody, AF7188, Affinity; Anti‐rabbit eIF2*α* antibody, #AF7688; Anti‐rabbit DDIT3/CHOP antibody, #AF6277, Affinity; 1:1000 anti‐rabbit ATF4 antibody, #DF6008, Affinity; 1:1000 Anti‐rabbit HMGB1 antibody, #AF7020, Affinity; 1:1000 anti‐rabbit Calreticulin antibody, Abconol, #A1066; 1:1000 Anti‐mouse NLRP3 antibody, Affinity, #BF8029; 1:1000 Anti‐rabbit TMS1/ASC antibody, #DF6304, 1:1000 Anti‐rabbit NF*κ*B p65 antibody, #AF5006; 1:1000 Anti‐rabbit Phospho‐NF*κ*B p65 (Ser536) antibody, #AF2006, Anti‐rabbit Caspase 1 antibody, #AF5418; Anti‐rabbit Cleaved‐Caspase 1 antibody, #AF4022; Anti‐mouse IL1 *β* monoclonal antibody, #BF8021; Anti‐rabbit Cleaved‐IL1 *β* antibody, #AF4006, anti‐rabbit Nrf2 antibody, #AF0639; Anti‐rabbit Phospho‐Nrf2 (Ser344) antibody, #AF7404) overnight at 4 °C temperature. Then, proteins were visualized using an HRP‐conjugated anti‐rabbit or anti mouse IgG (1:2000; Cell Signaling Technology) in combination with the ECL chemiluminescence system (Thermofisher).

### Statistical Analysis

Image J software (National Institutes of Health, Bethesda, MD, USA) was used to analyze microscopy data. Immunohistochemical A*β*1‐42 were counted in two fields on three tissue sections for each animal at a magnification of X400 (Nikon). Immunofluorescence intensities were averaged in two fields on three sections for each animal at a magnification of X600 (Leica), and cell numbers were summed in two fields on two sections for each animal at a magnification of X600 (Leica). SPSS software for Windows, version 19.0 (SPSS, Chicago, USA) or Prism 8.0 software (GraphPad, La Jolla, CA, USA) was used for statistical calculations. Two‐way Analysis of Variance (ANOVA) analysis of variance with a *Bonferroni's* post – hoc test for multiple comparisons was used to analyze two‐photon imaging data, and one‐way ANOVA analysis of variance with a *Bonferroni's* post‐hoc test for multiple comparisons was used to analyze other data. All data were expressed as the mean ± standard deviation (S.D). A value of *p < *0.05 was considered statistically significant.

### Ethic Approval Statement

This study was approved by Guangdong Laboratory Animal Monitoring Institute (A‐IACUC2022115), Guangzhou, China.

## Conflict of Interest

The authors declare no conflict of interest.

## Supporting information

Supporting Information

## Data Availability

The data that support the findings of this study are available from the corresponding author upon reasonable request.
