# Supplementary material for: Blood Brain Barrier‐Crossing Delivery of Felodipine Nanodrug Ameliorates Anxiety‐Like Behavior and Cognitive Impairment in Alzheimer's Disease
Source: Adv Sci (Weinh). 2024 Jul 9;11(34):2401731. doi: 10.1002/advs.202401731 (PMC11425895; doi:10.1002/advs.202401731)
Supplement: Supplementary file 1 — Supporting Information [file ADVS-11-2401731-s001.docx]

**Supporting Information**

**Blood brain barrier-crossing delivery of felodipine nanodrug ameliorates anxiety-like behavior and cognitive** **impairment in Alzheimer’s disease**

Xiaofei He^1#^, Yuan Peng^2#^, Sicong Huang^3#^, Zecong Xiao^4^, Ge Li^5^, Zejie Zuo^1^, Liying Zhang^1^, Xintao Shuai^4*^, Haiqing Zheng^1*^, Xiquan Hu^1*^


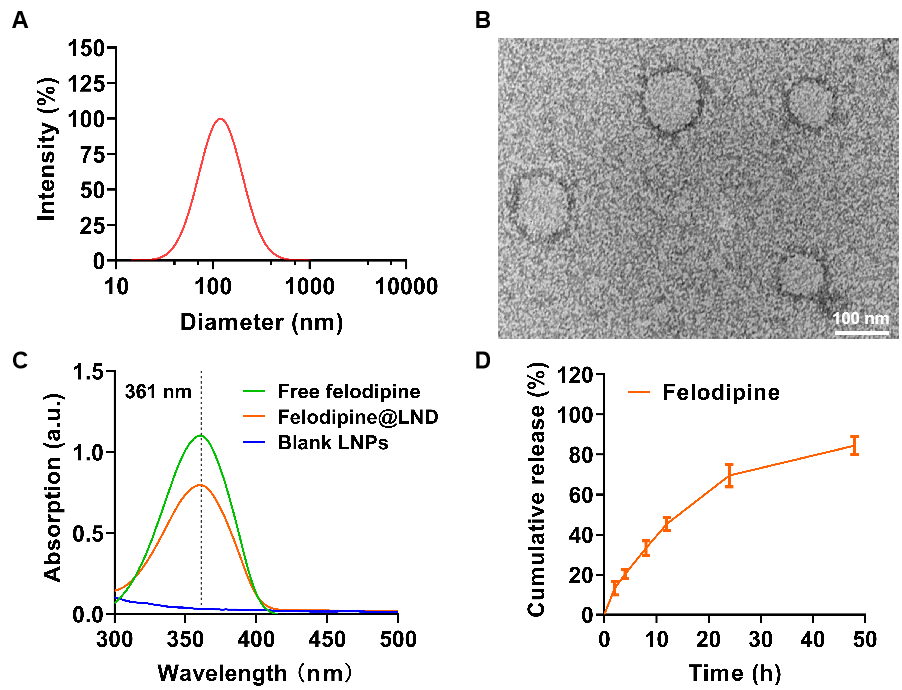


**Figure S1.** Characterization of felodipine@LND. (A) Size distribution of felodipine@LND. (B) TEM images of felodipine@LND. (C) UV absorption spectra for free felodipine, felodipine@LND and blank LNPs. (D) In vitro release profile of felodipine from felodipine@LND in PBS of pH 7.4 at 37 °C (n = 3; means ± SD).


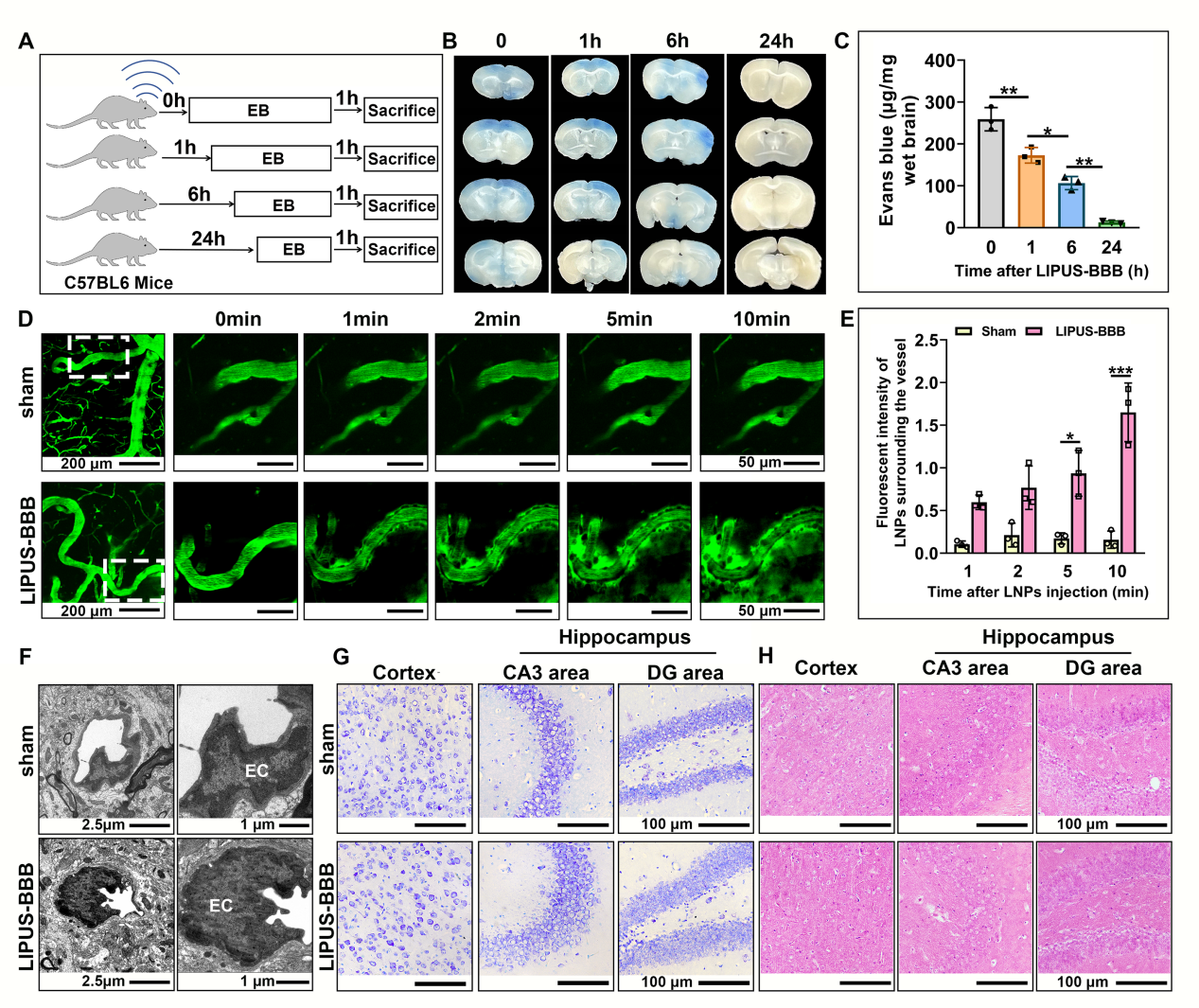


**Figure S2. Low-intensity pulsed ultrasound (LIPUS) reversibility opened the blood brain barrier (BBB) and facilitated the LNPs permeation in brain parenchyma.** A) Experimental design for verification for LIPUS - BBB opening. B). Evans blue staining at 0 h, 1 h, 6 h and 24 h after LIPUS. C) Comparison analysis with evans bule staining. (D-E) Two-photon imaging and comparison analysis for coumarin 6@LNPs permeation in brain parenchyma at different times after tail intravenous injection of coumarin 6@LNPs between sham and LIPUS-BBB groups. F) Transmission electron microscope (TEM) for cerebral vessels in sham and LIPUS-BBB group. G) Nissl's staining of the neurons of mice in the sham and LIPUS-BBB groups. H) hematoxylin and eosin (H&E) staining for extravasation of erythrocytes in sham and LIPUS-BBB group (*n* = 3; means ± SD; *****p* < 0.0001, ****p* < 0.001, ***p* < 0.01,**p* < 0.05).


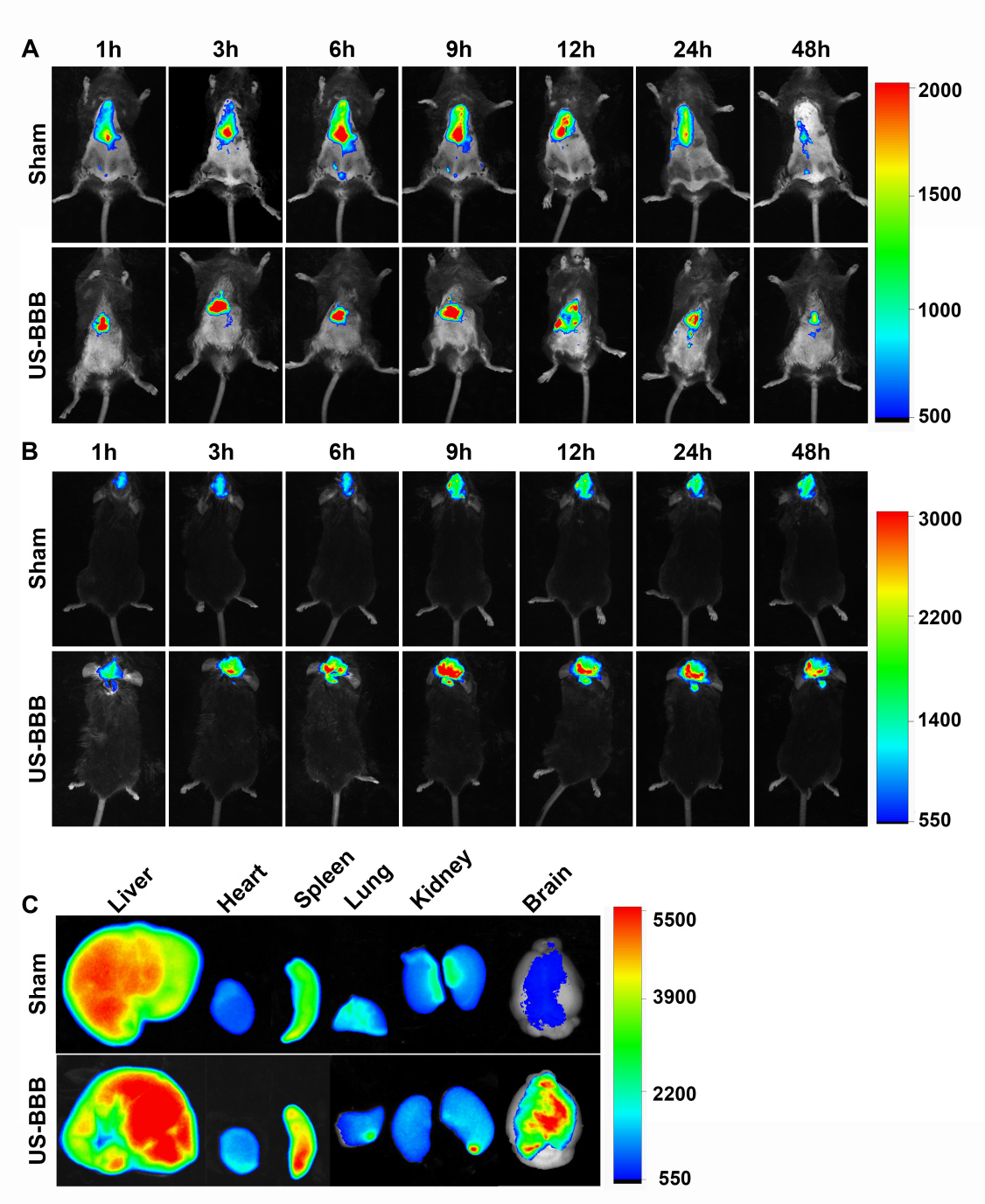


**Figure S3.** A) Real time fluorescence imaging of organs from mice with or without US-BBB after intravenously injection of DiR@LNPs. B)Real time fluorescence imaging of brain at different time after tail vein injection of DiR@LNPs. C) Ex vivo fluorescence imaging of Livers, hearts, Spleens, Lungs, Kidneys and brains from mice with or without US-BBB.


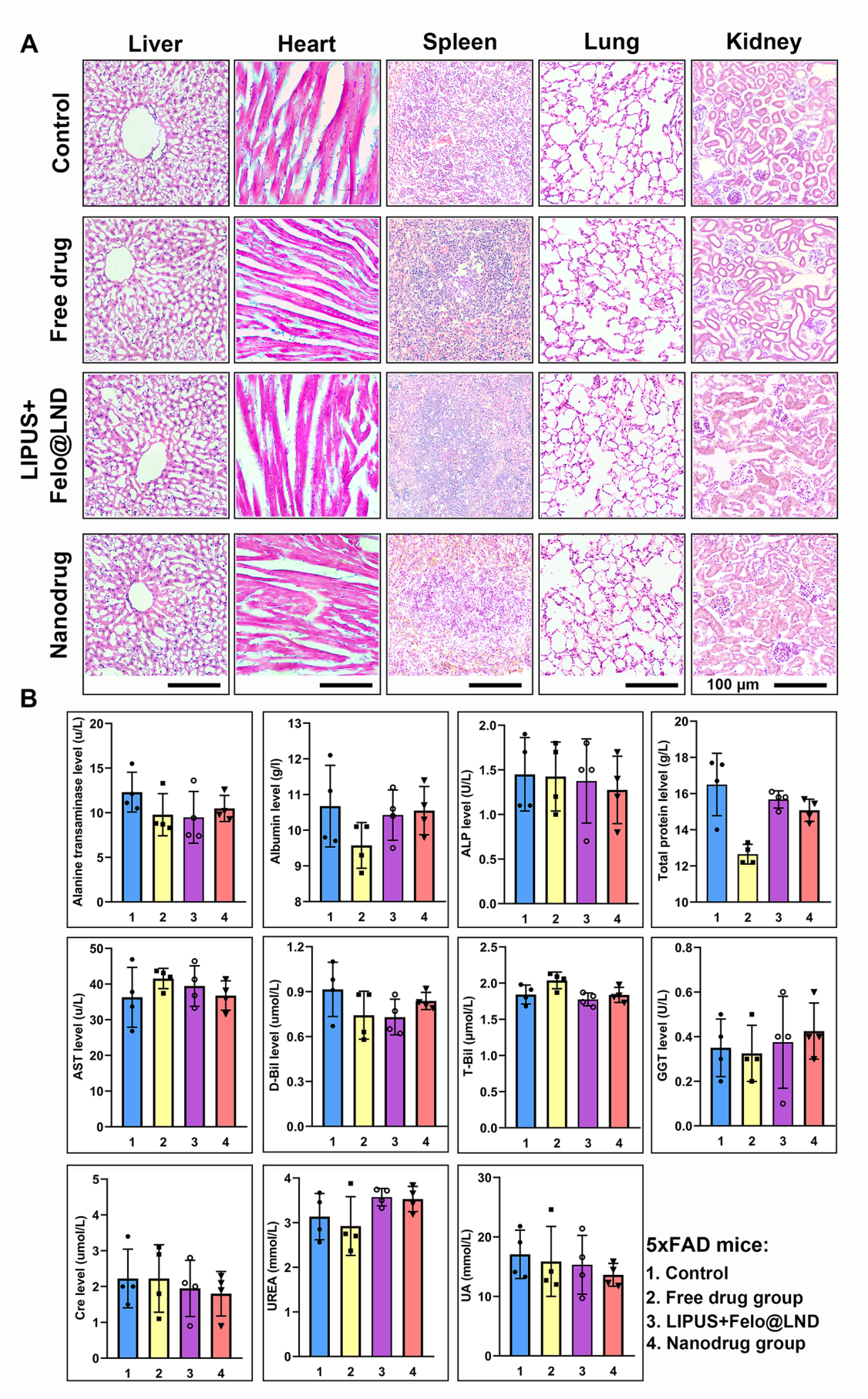


**Figure S4.** **Felodipine@LND injections showed no toxicity to** **systemic organ.** A) HE staining for the Liver, Heart, Spleen, Lung and Kidney showcasing the histological safety of Felodipine@LND. B) Serological examination of hepatic function and renal function (samples diluted three-fold). (*n* = 4; means ± SD; *****p* < 0.0001, ****p* < 0.001, ***p* < 0.01,**p* < 0.05)
